# Stretchable Filler/Solid Rubber Piezoresistive Thread Sensor for Gesture Recognition

**DOI:** 10.3390/mi13010007

**Published:** 2021-12-22

**Authors:** Penghua Zhu, Jie Zhu, Xiaofei Xue, Yongtao Song

**Affiliations:** 1 School of Computer, North China Institute of Aerospace Engineering, Langfang 065000, China; zhuph@nciae.edu.cn (P.Z.); xxf32313@nciae.edu.cn (X.X.); songyongtao@nciae.edu.cn (Y.S.); 2 Aerospace Software Joint Innovation Center, North China Institute of Aerospace Engineering, Langfang 065000, China

**Keywords:** stretchable piezoresistive sensor, Ag@GMs/SR composite, high gauge factor, low hysteresis, gesture recognition

## Abstract

Recently, the stretchable piezoresistive composites have become a focus in the fields of the biomechanical sensing and human posture recognition because they can be directly and conformally attached to bodies and clothes. Here, we present a stretchable piezoresistive thread sensor (SPTS) based on Ag plated glass microspheres (Ag@GMs)/solid rubber (SR) composite, which was prepared using new shear dispersion and extrusion vulcanization technology. The SPTS has the high gauge factors (7.8~11.1) over a large stretching range (0–50%) and approximate linear curves about the relative change of resistance versus the applied strain. Meanwhile, the SPTS demonstrates that the hysteresis is as low as 2.6% and has great stability during 1000 stretching/releasing cycles at 50% strain. Considering the excellent mechanical strain-driven characteristic, the SPTS was carried out to monitor posture recognitions and facial movements. Moreover, the novel SPTS can be successfully integrated with software and hardware information modules to realize an intelligent gesture recognition system, which can promptly and accurately reflect the produced electrical signals about digital gestures, and successfully be translated into text and voice. This work demonstrates great progress in stretchable piezoresistive sensors and provides a new strategy for achieving a real-time and effective-communication intelligent gesture recognition system.

## 1. Introduction

As flexible electronics continue to develop in reliability and comfort, the applications of stretchable and wearable sensing electronics in biomechanical detection and gesture recognition have attracted the attention of many scholars [1,2,3,4]. Up to now, several researchers have explored a variety of stretchable sensors with various principles (triboelectricity [5,6,7], piezoelectricity [8,9] and piezoresistance [10,11,12], etc.) to monitor signals generated by human movements or physiological characteristics. Among them, the stretchable, cost-effective, light-weight sensors based on triboelectric effect can be acted as a self-powered sensor to achieve sustainable power while identifying human postures. However, the triboelectric sensors are sensitive to the environmental parameters (such as temperature, humidity and air pressure) that have been confirmed by the researches of Zhong Lin Wang team [13] and Su et al. [14], which could lead to the lack of stability in output performances. The stretchable piezoelectric sensors with positive piezoelectric effect are active in response to the tensile force, however, the complex preparation process and the depolarization of piezoelectric material will seriously hinder their wide applications. Comparatively, the stretchable piezoresistive sensors have the advantages of high reliability, low cost, and simple fabrication, which makes them provide promising candidates for the development of increasingly diversified stretchable electronics.

To realize the stretchable piezoresistive sensor, the carefully designed structures and the appropriate elastic piezoresistive materials are two key points [15,16,17,18]. In terms of structure design, the flexible piezoresistive sensors are fabricated into pleats or serpentine structures to complete the stretchability of the device [19,20]. However, the single tensile direction and tiny strain limit their applications in human posture recognition systems. In the selection of stretchable piezoresistive materials, stretchable sensitive composites with conductive fillers embedded in elastic matrix material are very popular. When choosing an elastic matrix, the liquid polymers (such as polydimethylsiloxane (PDMS) [21,22,23], silicone rubber [24,25], polyurethane (PU) [26,27,28,29], etc.) are the most promising candidate for the field of stretchable piezoresistive sensors due to their remarkable elasticity, mechanical stability and high repeatability. The stretchable piezoresistive composites are usually achieved by mechanically stirring the conductive materials into liquid polymers. However, the stirring and rotating force is not enough to break the strong viscosity of the polymer and the large filler agglomeration, resulting in the cluster effect of the nanomaterials. Thus, the solvents such as methylbenzene with inherent hyper-toxicity are always added to obtain a uniformly distributed composite, however, the sensors formed in this way are not suitable for long-term configuration on the skin and human joints. Therefore, exploring a stretchable piezoresistive sensor based on the homogeneous dispersion of fillers in hyper-elastomer matrix plays an extremely important role in the development of stretchable electronics.

Herein, we demonstrate a simply and easily well-dispersed route for implementing a stretchable piezoresistive thread sensor (SPTS) consisting of Ag plated glass microspheres (Ag@GMs) and solid rubber (SR). The preparation process of the SPTS breaks the bottleneck of difficult dispersion of filler agglomerates in traditional liquid polymer matrix by using new shear dispersion and new shear dispersion and extrusion vulcanization technology. The SPTS exhibits that the averaged gauge factors (GF) are about 7.8, 8.1 and 11.1 in three linear increasing stages of 0–20%, 20–40%, and 40–50%. Meanwhile, the SPTS based on the quantitative investigations shows superior performances such as favorable stretchability, low hysteresis, and excellent durability. The SPTS can be easily assembled to human body to recognize postures and facial movements. Moreover, the SPTS was combined with the conversion circuit, microprocessor control circuit, the serial communication circuit and the terminal display software to form an intelligent gesture recognition system, which can promptly and accurately reflect the produced electrical signals about digital gestures, and successfully be translated into text and voice. Thus, the novel SPTS has great potential in developing the next generation of intelligent sign language recognition electronics.

## 2. Results and Discussion

**Figure 1:**
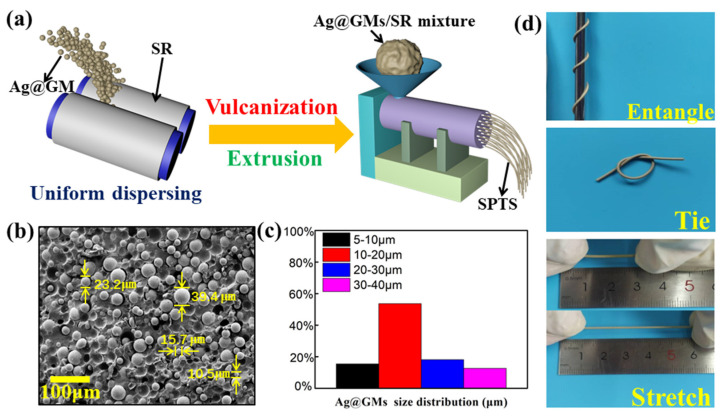
(**a**) The preparation process of the stretchable piezoresistive thread sensor (SPTS). (**b**) The SEM images of the SPTS. (**c**) The size distribution diagram of the Ag@GMs. (**d**) The various states of the SPTS were demonstrated including entangled, tied, and stretched.

To obtain a stretchable piezoresistive sensor, proper materials selection plays an important role in our work. The SR with the super-elasticity and tough property was selected as the matrix material; as for the functional material, the low-cost Ag@GMs was selected. It’s worth noting that the shear dispersion process instead of the original mechanical stirring method enhances the ability to destroy clusters, resulting in evenly distributed piezoresistive composite. As shown in Figure 1a, two rollers with different rotating speeds provide strong shear force and extrusion pressure, which can overcome the van der Waals forces between the conductive fillers and the strong adhesion of SR, well solving the serious problem caused by uneven dispersion of the functional fillers in the matrix. Especially note that the “triangle package” method was used to accelerate the dispersion of the packed phase in the matrix in the process of dispersing the fillers [30]. Finally, the polymer chain of SR was crosslinked with the curing agent at high temperature and high pressure, and the mixture was extruded into a stretchable piezoresistive thread sensor with a diameter of 1 mm. Significantly, in the preparation of stretchable piezoresistive sensor, it is necessary to consider that the increase of the filling amount of AG@GMs will hinder the tensile property of the sensor. According to our previous manufacturing experience about fillers/SR composite [31,32], the mass ratio of AG@GMs and SR was selected as 3:1. Figure 1b shows the scanning electron microscopy (SEM) image of the SPTS composed of AG@GMs (75 wt%) and SR. Obviously, Ag@GMs were evenly embedded into SR to form a compact three-dimensional network structure. Moreover, as shown in Figure 1c, the size distribution of Ag@GMs can be collected from Figure 1b, which indicates that the diameter is mainly distributed in 5~40 μm. In addition, Figure 1d indicates the SPTS entangled, tied, and stretched, clearly illustrating the flexibility and robustness of the fabricated piezoresistive sensor.

**Figure 2:**
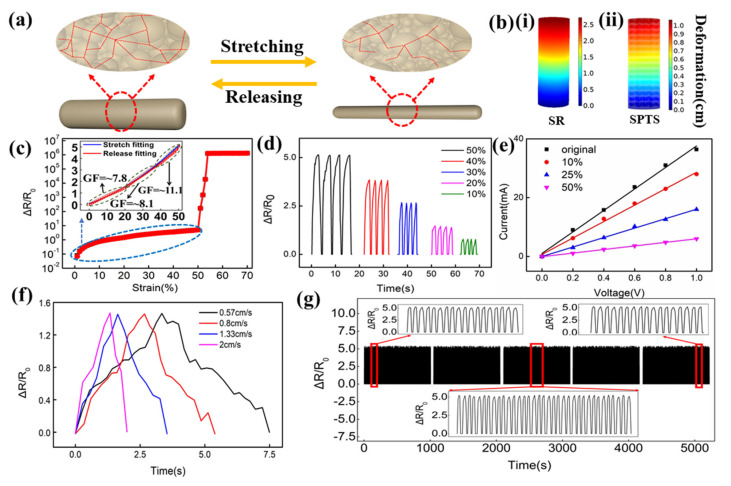
(**a**) The tensile-resistance sensing mechanism of the SPTS. (**b**) The strain distributions for the SR and SPTS being stretched force. (**c**) Curves of relative change in resistance against the different stretched and released strains. (**d**) Responsive behaviors of the SPST at different stretching amounts. (**e**) The current-voltage curves of the SPTS for tensile strain in the range of 0–50%. (**f**) The correlation between the relative variation in resistance versus tensile speeds. (**g**) Stability test during 1000 cycles at operating strain of 50%.

The tensile-resistance sensing mechanism of the sensitive material has been studied in-depth, as shown in Figure 2a. The stretched/released force applied to the sensing component produces reversible mechanical deformation of the SPTS. The application of tensile force causes deformation of the SPTS in the same direction as the force, leading to the reassembly of Ag@GMs in SR. The number of Ag@GMs in the unit length is reduced and the conductive path of the SPTS changes. That is, the connection points between the fillers become less, thereby increasing the resistance value of the SPTS. To further shed light on the relationship between stretching force and sensitive material, the finite element analysis (FEA) was conducted using the COMSOL software. Figure 2b shows the strain distributions for the SR and SPTS being stretched force. The SR was modeled as a cylinder (Figure 2(bi)), and the SPTS was constructed to contain uniformly distributed conductive particles within the SR (Figure 2(bii)). The fixed constraints were applied on the bottom faces and 100 N/m^2^ tension was applied on the top faces along the positive direction of *Z*-axis in the two models, respectively. Comparatively, the deformation of Ag@GMs/SR composite is smaller than that of SR, probably due to the much higher Young’s modulus of Ag@GMs than that of SR. Meanwhile, it can also be seen that the stress on the upper part varies greatly along the direction of tension. In this case, the larger the separation distance between the Ag@GMs at the upper part will result in a change in the conductive path of the sensitive unit.

To analyze the electrical and mechanical properties of the SPTS, the systematic experiments have been performed. As shown in Figure 2c of the revised manuscript, the tensile-sensing mechanism of the sensitive material has been studied in depth. It can be seen that when the strain exceeds 50%, the ΔR/R_0_ will raise greatly, and then ΔR/R_0_ tends to be stable with the increase of the strain. At this time, there are almost no connection points between AG@GMs, resulting in almost non-existent conductive path. Therefore, the SPTS was selected within 50% strain for quantitative performance test. The inset of Figure 2c shows that the averaged GF of the sensor in three linear increasing stages of 0–20%, 20–40%, and 40–50%. The GF values are achieved by

(1) $$\mathrm{GF}=\left[\left(R-R_{0}\right)/R_{0}\right]/\mathrm{strain}$$

where R and R_0_ are the current resistance and the initial resistance, respectively, and strain is the stretching amount in the longitudinal direction. The GF values were calculated as 7.8, 8.1, and 11.1, respectively. Furthermore, the hysteresis (Hy) of the SPTS was studied by measuring the relative resistance change of the stretching/releasing cycles within 50% strain. The $H_{y}$ is expressed as

(2) $$H_{y}=\frac{S_{\mathrm{stretch}}-S_{\mathrm{release}}}{S_{\mathrm{stretch}}}\times 100\%$$

where $S_{\mathrm{stretch}}$ and $S_{\mathrm{release}}$ are the integral areas of the stretch and release curves, respectively. In this work, the hysteresis of the SPTS was as low as 2.6%, and the relative change of resistance in the release process was slightly less than that in the tensile process. This phenomenon may be explained by the slippage of Ag@GMs under stretching and the delay time associated with the hysteresis of elastic matrix material [33,34]. As shown in Figure 2d, the SPTS can reliably distinguish the dynamic response of different stretching amounts. Figure 2e describes the current-voltage curves of the SPTS for tensile strain in the range of 0–50%. Obviously, the current was an almost linear function of voltage under different tensile constants, and the slope of curve was reduced with the increase of the strain. In other words, the resistance becomes large as the strain increases, which may be due to the widening of the gap between the conductive particles with the enlarging of the tensile amount. Considering that the SPTS needs to respond to different strain rates in practical applications, the piezoresistive behaviors of the SPTS were obtained at different stretching speeds (Figure 2f). It can be seen that SPTS has stable piezoresistive response and the sensitivity of the SPTS is independent of strain rate. The durability test of the SPTS was performed under 1000 stretching/releasing cycles at 50% strain, as shown in Figure 2g. Notably, the SPTS has long-term stretching/releasing durability and outstanding mechanical property.

**Figure 3:**
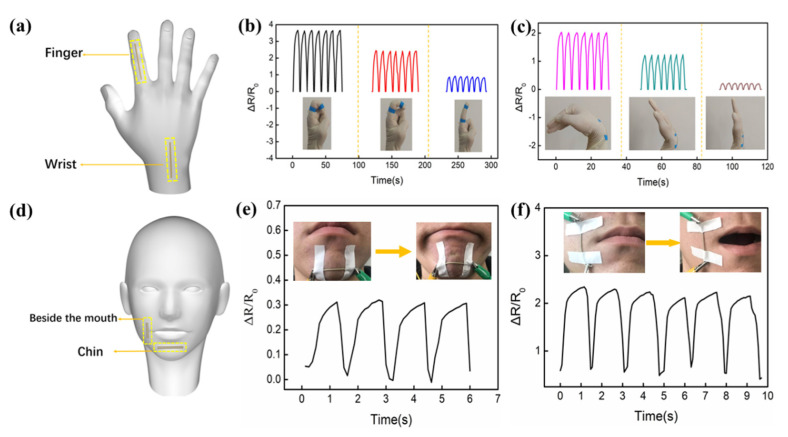
(**a**) The SPTS was fixed on a human finger and wrist for posture recognition. Dependence of the acquired electrical signals on the bending angles of (**b**) finger and (**c**) wrist. (**d**) The SPTS was attached on the facial skin for investigating relevant dynamic facial expression. Acquired electrical signals from (**e**) chin moving and (**f**) mouth opening.

Given the excellent stretchability of the fabricated SPTS, it can be comfortably assembled to human fingers and wrists for posture recognition, as shown in Figure 3a. The SPTS was fixed on a tester’s finger knuckle to respond to different bending-releasing motion states (Figure 3b). The output reliability of the sensing unit was identified by seven times continuous repeating experiments for each bending angle. Clearly, the output signals increase with the enlarging of bending angle, namely, the electrical signals are confirmed by the magnitudes of the stretching motions. Similarly, the SPTS was installed on the wrist utilizing the output electrical signals to distinguish different bending angles, as shown in Figure 3c. Additionally, the wearable SPTS was directly attached to the facial skin for investigating dynamic facial expressions (Figure 3d). As shown in Figure 3e,f, the SPTS can respond to chin and mouth movements, indicating that it has considerable potential for real-time monitoring and quantitative assessments of complex facial expressions.

**Figure 4:**
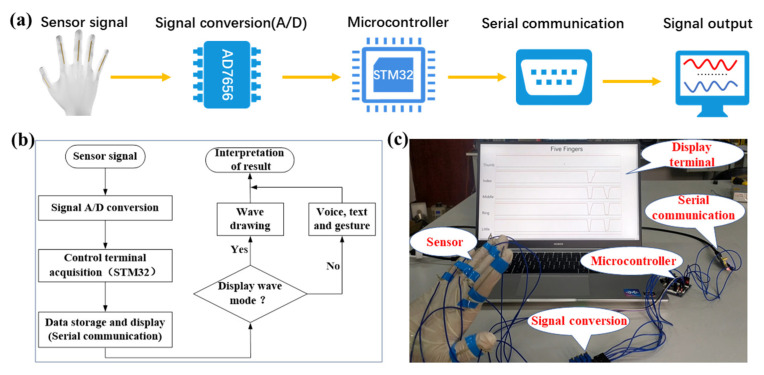
The SPTS-based intelligent sign language translation system. (**a**) Implementation scheme. (**b**) Flow chart. (**c**) Actual photograph.

Due to outstanding advantages of electrical and mechanical performances, the SPTS could be widely explored in the area of sensor systems and wearable electronics. The SPTS was combined with the conversion circuit, microprocessor control circuit, the serial communication circuit and the terminal display software to form an intelligent gesture recognition system, as depicted in Figure 4a. The flow chart of hardware acquisition and software display based on SPTS is shown in Figure 4b. Firstly, the analog signal of the SPTS was converted into digital signal by six-channels AD7656 module, then the acquisition and transmission were controlled by an STM32 microcontroller, and finally the waveform display or gesture recognition of the collected data was carried out on the upper computer. The overall test diagram about the SPTS-based intelligent gesture recognition system was shown in Figure 4c. Overall, this work was integrated by the sensitive component, the software and hardware information systems to realize the acquisition and display of finger tensile stress, which is expected to promote the development of wearable sign-to-speech translation system.

**Figure 5:**
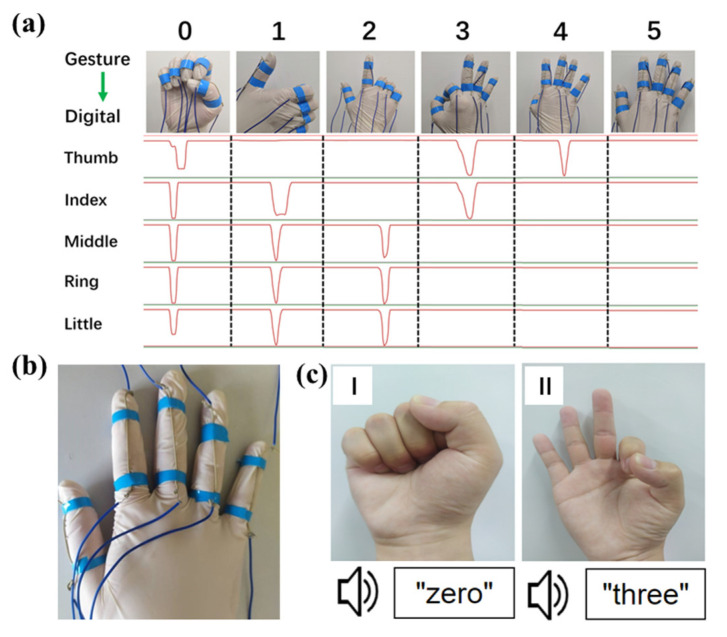
(**a**) Output distributions of electrical signals corresponding to five digital gestures (0, 1, 2, 3, 4 and 5). (**b**) Photograph of the five fingers completely extended initial state. (**c**) Gestures of 0 and 3 were translated into text and voice.

To further demonstrate the real-time gesture recognition, five digital gestures (0, 1, 2, 3, 4 and 5) were selected, and the output distributions of electrical signals corresponding to digital gestures were shown in Figure 5a. The initial state was set to five fingers completely extended state (Figure 5b). When a number is implemented, the fingers need to be bent, and the corresponding channels will have a waveform appearance. To enhance the user experience, the gesture pictures of the gesture recognition system were converted into text and voice. As shown in Figure 5c and Supplementary Materials Video S1, the digital numbers were successfully translated into text and voice, and each gesture was also displayed on the main interface. These demonstrations indicate the great potential of the SPTS in developing the next generation of intelligent sign language recognition.

## 3. Conclusions

In summary, the stretchable Ag@GMs/SR composite was manufactured to solve the homogeneous mixing issue about the elastic filler-polymer piezoresistive sensor, owing to the existence of van der Waals forces of conductive fillers and viscosity of polymer. The SPTS not only exhibits the high stretchable capacity of 50% strain, but also has the high gauge factors of 7.8, 8.1 and 11.1 in three linear increasing stages (0–20%, 20–40%, and 40–50%). In addition, the SPTS demonstrates that the hysteresis is as low as 2.6% and has great stability during 1000 stretching/releasing cycles at 50% strain. The SPTS with mechanical and electrical robustness has been adopted as an effective sensing unit to realize posture recognitions and facial movements. Furthermore, the SPTS was combined with the modules of hardware acquisition and software display to form an intelligent gesture recognition system for displaying signal waveforms and converting gestures into text and voice. This work is expected to provide a new communication way between the disabled (such as blind, deaf, mute people, etc.) and the normal people, enhancing the quality of life of people with disabilities.

## 4. Experimental Section

### 4.1. Fabrication of the Stretchable Piezoresistive Sensor

Firstly, the SR and the curing agent (TN 920, Dongguan Tian Silicone rubber Science and Technology Co., Ltd., Dongguan, China) with a mass ratio of 100:3 were placed between two rollers of the silicon mixing machine for 5 min, so that the curing agent can be completely contacted with SR. Then, the Ag@GMs (W-6, Shenzhen Effecte Science and Technology Co., Ltd., Shenzhen, China) were poured into the stacked SR above the middle of two rollers at a mass ratio of 3:1 and drawn into the two rollers with the SR. The careful design about the two roll spacing (500 μm), roll speed (130 rad/s) and two roll speed ratio (1:1.2) can provide large laminar shear force and extrusion pressure. Significantly, after mixing and dispersion for a while, the mixture only flows axially with the drum, so it is necessary to cut the mixture on the rollers every 5 min and continue mixing in by triangular wrapping. After mixing for 1 h, the Ag@GMs/SR composite was vulcanized and extruded by an extrusion machine (SY-6217, Dongguan Shiyan Precision Instrument Co., Ltd., Dongguan, China) at 110 °C to obtain a stretchable piezoresistive thread sensor with the diameter of 1 mm.

### 4.2. Measurement System

The uniform mixing process of Ag@GMs and SR was carried out on the roller of the silicon mixing machine (XH-401, Dongguan Xihua Testing instrument Co., Ltd., Dongguan, China). The microstructures of the sample were characterized by the field emission scanning electron microscopy (ZEISS EVO18, Carl Zeiss Jena, Baden-Württemberg, Germany). The output electrical signals were recorded by a KEITHLEY 2611B system electrometer. The tensile test was conducted by the HP series digital display tension meter (Adeborg Instruments Co., Ltd., Jiaxing, China).

## Supplementary Materials

The following supporting information can be downloaded at: https://www.mdpi.com/article/10.3390/mi13010007/s1, Video S1: Demonstration of intelligent gesture recognition system.
